# Gaze Behavior in a Natural Environment with a Task-Relevant Distractor: How the Presence of a Goalkeeper Distracts the Penalty Taker

**DOI:** 10.3389/fpsyg.2018.00019

**Published:** 2018-01-26

**Authors:** Johannes Kurz, Mathias Hegele, Jörn Munzert

**Affiliations:** Neuromotor Behavior Laboratory, Department of Psychology and Sports Science, Justus-Liebig-University, Germany

**Keywords:** far-aiming task, natural environment, gaze behavior, performance, penalty

## Abstract

Gaze behavior in natural scenes has been shown to be influenced not only by top–down factors such as task demands and action goals but also by bottom–up factors such as stimulus salience and scene context. Whereas gaze behavior in the context of static pictures emphasizes spatial accuracy, gazing in natural scenes seems to rely more on where to direct the gaze involving both anticipative components and an evaluation of ongoing actions. Not much is known about gaze behavior in far-aiming tasks in which multiple task-relevant targets and distractors compete for the allocation of visual attention via gaze. In the present study, we examined gaze behavior in the far-aiming task of taking a soccer penalty. This task contains a proximal target, the ball; a distal target, an empty location within the goal; and a salient distractor, the goalkeeper. Our aim was to investigate where participants direct their gaze in a natural environment with multiple potential fixation targets that differ in task relevance and salience. Results showed that the early phase of the run-up seems to be driven by both the salience of the stimulus setting and the need to perform a spatial calibration of the environment. The late run-up, in contrast, seems to be controlled by attentional demands of the task with penalty takers having habitualized a visual routine that is not disrupted by external influences (e.g., the goalkeeper). In addition, when trying to shoot a ball as accurately as possible, penalty takers directed their gaze toward the ball in order to achieve optimal foot-ball contact. These results indicate that whether gaze is driven by salience of the stimulus setting or by attentional demands depends on the phase of the actual task.

## Introduction

One of the most prominent puzzles in vision science concerns how the oculomotor system determines where to look in complex natural environments. Previous research on the selection of gaze fixations has generated extensive empirical evidence in support of a two-component framework consisting of a fast, preattentive mechanism that biases an observer’s attention toward highly salient stimuli in the environment in a bottom–up fashion and a second, slower, top–down mechanism that can apply all kinds of criteria to select environmental stimuli in a flexible, cognitively controlled fashion. A common conceptualization of the first mechanism is based on the notion of a saliency map (e.g., Koch and Ullman, 1985; Itti and Koch, 2000). This is a two-dimensional representation of the visual environment in which the most salient object corresponds to a single location that determines the next target of oculomotor action in a winner-take-all manner. Despite the popularity of this saliency map approach when explaining how the oculomotor system selects fixation targets, previous research has estimated the accuracy of predicting the target location of gaze fixations correctly to be only 57 to 68% (Itti and Koch, 2000; Kienzle et al., 2009). Even though prediction accuracy has been shown to benefit from taking oculomotor strategies into account (e.g., center bias, see Tatler and Vincent, 2009), there is still a considerable proportion of fixations that cannot be explained on the basis of stimulus salience alone. In addition, even though low-level featural information, which defines the saliency map of an image or a scene, has been shown to correlate with the location of fixations in complex, natural scenes as well (Borji et al., 2013), it is unclear whether such a correlation indeed reflects a causal relationship between stimulus salience and fixation selection in human observers.

A second influential research tradition has focused on investigating how ongoing goal-directed behavior influences the control of eye movements (see Hayhoe and Ballard, 2005, for a review). Gaze behavior has been studied during everyday activities such as making a peanut butter sandwich (Hayhoe et al., 2003) or preparing a cup of tea (Land et al., 1999) as well as during highly dynamic sports activities such as playing squash (Hayhoe et al., 2012), cricket (Land and McLeod, 2000; Mann et al., 2013), or soccer (Noël and van der Kamp, 2012; Timmis et al., 2014). These studies have shown that humans fixate almost exclusively on task-related locations in their environment, suggesting that salience-based mechanisms are inhibited or even switched off when engaging in goal-directed behavior (Schütz et al., 2011). However, several studies have shown that differences in task demands influence gaze behavior even when either no task instructions are given or task instructions remain identical. In a study by Pelz and Rothkopf (2007), subjects had to walk on different types of walkways (paved and dirt path). Although instructions were the same for both tasks, gaze behavior differed: subjects directed only 35% of their gaze onto the paved path, but 62% onto the dirt path. Task demands do not just influence gaze behavior; they also influence head movements and gait (Marigold and Patla, 2008). When subjects had to walk on a multisurface terrain, their downward head pitch angle increased and both their gait speed and step length decreased when the lower visual field was blocked compared to when the lower visual field was not blocked. These different patterns of gaze behavior and head movements were also found when the terrain was made more irregular (’t Hart and Einhäuser, 2012). Similar to walking, real-life cycling on different types of terrains also led to differences in gaze behavior (Zeuwts et al., 2016). Gaze was directed more toward the path when cycling on a dirt path (71.9%) compared to a paved path (24.1%). These results indicate that gaze behavior is highly task-specific, and that the demands of the task seem to play an important role.

Another problem with most empirical studies that adopt a salience-based approach to analyzing gaze behavior has been their focus on studying observers who passively view a static picture or a visual scene. In everyday life, however, humans actively interact with their environment in a goal-directed fashion (Patla and Vickers, 2003; ’t Hart and Einhäuser, 2012). Hence, it would seem to be essential to analyze gaze behavior in natural environments. Indeed, several studies found differences in the pattern of gaze behavior between laboratory and real-world tasks. For example, Dicks et al. (2010) compared gaze behavior of soccer goalkeepers in an artificial and a natural environment. Results showed different gaze behavior in the two conditions. In particular, there were earlier fixations and longer durations of fixation on the ball in a natural environment. Studies on walking in real life compared to watching videos of walking also found significantly different patterns of gaze behavior (’t Hart et al., 2009; Foulsham et al., 2011). Foulsham et al. (2011) reported that in real life, eyes were focused more centrally due to participants making head movements instead of large saccades to direct their gaze toward different relevant locations. In real life, subjects directed their gaze more toward near objects and toward the path they were walking on, whereas subjects watching a video directed their gaze more toward far objects such as lampposts, trees, and distant buildings and less toward the walking path. A study by ’t Hart et al. (2009) revealed that in the laboratory, the display influences gaze, because the visual angle is smaller than that in real life. They also mentioned that restricting the head movements while watching videos leads to limitations in gathering further information (e.g., vestibular and other crossmodal information). These findings are in line with a study by Pelz et al. (2001) who found that eye-head coordination is a synergetic linkage rather than an obligatory one. In laboratory tasks, these synergetic effects are mostly interrupted, and this can result in a different pattern of gaze behavior. In the laboratory, walls are often blank or only a few fixation points are present, whereas in real life, many feasible fixation points are present, and these fixation points are also changeable (Pelz and Rothkopf, 2007). Similar to results reported by Foulsham et al. (2011), Zeuwts et al. (2016) found that gaze was directed more toward the path during real-life cycling (48.6%) than in laboratory tasks (29.8%). In this study, subjects had to cycle on a paved and on a dirt path in both a real-life and a laboratory setting. Results for cycling on a dirt path revealed higher similarities between real-life and laboratory than on a paved path. In contrast, face-detection studies (Peterson et al., 2016) have shown that results gained in the laboratory are also comparable with those in real life. This all suggests that under certain task constraints (e.g., face detection, increasing task complexity), gaze behavior in the laboratory can predict gaze behavior in real life (Peterson et al., 2016; Zeuwts et al., 2016).

In addition, gaze behavior during the observation of static pictures or scenes differs from that during dynamic interactions with the natural environment. The former seems to emphasize spatial accuracy, whereas the latter seems to emphasize where to direct the gaze (Land and McLeod, 2000). The difference is due to “just-in-time” mechanisms that rely on an information pick-up at the point in time required for the task (Ballard et al., 1995). This supports the notion that the timing of gaze during ongoing interactions with the environment is tuned actively to the instantaneous demands of action control in a top–down manner rather than passively reflecting the influence of the bottom–up processing of stimulus properties (Hayhoe and Ballard, 2005). Indeed, observers will fixate on an empty location in the environment such as where they intend to place a cup or aim to intercept a ball—and this can hardly be explained on the basis of stimulus salience. In a rather elegant study, Nuthmann and Henderson (2010) showed that gaze is directed toward the center of objects and that attention is object-based while watching a scene. One of their main findings was to show that this gaze pattern is found for real objects but only to a weaker degree for salience proto-objects. Tong et al. (2017) have also demonstrated that gaze while walking around is not driven mainly by stimulus salience. Notwithstanding this substantial evidence for a goal-directed influence on gaze behavior, not much is known about how the interaction between stimulus salience and task demands influences gaze behavior in tasks involving multiple potential targets for fixations that differ in stimulus salience and task relevance not only between but also within actions. The mechanisms driving these processes in natural environments are simply not fully understood. This holds specifically for task requirements that include more than one target in a dynamic context.

The penalty kick in soccer is a task containing multiple targets for fixations that differ in stimulus salience and task relevance. The penalty taker has to deal with two targets: a proximate and a distal target. On the one hand, the foot has to hit the ball (proximal target) as accurately and as forcefully as possible; on the other hand, the shot has to accelerate the ball toward a specific location within the goal (distal target). In addition, penalty situations contain a goalkeeper who tries actively to prevent the penalty taker from scoring a goal. To this end, goalkeepers not only seek to intercept the ball but also frequently try to distract the penalty taker before and during the penalty. Goalkeepers can thus be conceived as task-relevant distractors. They are task-relevant because their behavior directly influences the success or failure of the penalty taker’s action. They are distractors because they actively try to increase their salience in order to distract the penalty taker by waving their arms up and down, moving on the goal line (Wood and Wilson, 2010a; Navarro et al., 2013), or wearing a colored (e.g., red) jersey (Greenlees et al., 2008). In order to deal with the goalkeeper, previous research has identified three strategies for taking a penalty: (a) the keeper-independent strategy, (b) the keeper-dependent strategy (Kuhn, 1988; van der Kamp, 2006), and (c) as described by Wood and Wilson (2010b), the opposite-independent strategy. In the keeper-independent strategy, the penalty taker selects the target location to shoot toward before the run-up. During the run-up, she or he does not consider reactions made by the goalkeeper. The decision on where to aim depends on the penalty taker’s favorite kicking side or on the placement of the goalkeeper on the goal line (Masters et al., 2007; Noël et al., 2016). In contrast, in the keeper-dependent strategy, the penalty taker tries to obtain information from the goalkeeper’s reactions during the run-up. In the opposite-independent strategy, the penalty taker looks to one side of the goal and shoots to the opposite side, irrespective of reactions made by the goalkeeper.

Studies in a realistic setup have shown that penalty takers using the keeper-dependent strategy direct their gaze more toward the goalkeeper compared to the ball and the target location. In contrast, penalty takers using the keeper-independent strategy direct their gaze more toward the ball compared to the goalkeeper and the target location (Wood and Wilson, 2010a; Noël and van der Kamp, 2012). Timmis et al. (2014) showed that participants directed their gaze mostly toward the ball (∼60%) irrespective of whether they were taking power or placement penalty kicks. In contrast, studies in a laboratory setup (Bakker et al., 2006; Wilson et al., 2009; Binsch et al., 2010) have shown deviant results. In these studies, penalty takers direct their gaze mainly toward the goalkeeper or the target location irrespective of the strategy/instructions. These findings point to the limitations in the experimental setup (e.g., no or a one-step run-up, smaller goal size), thereby showing the importance of using a realistic setup when investigating gaze behavior.

Up to now, it is unclear how far task-dependent fixation selection is able to prevent the fixation of salient distractor stimuli in the environment during the execution of goal-directed actions. Assuming that oculomotor control, that is, the selection of fixation targets in the course of executing an action, is task-specific and attuned on a moment-to-moment basis to the requirements of the task at hand, we hypothesize that a salient distractor will influence gaze behavior differentially over the course of an action. Furthermore, we expect that the extent to which gaze behavior is affected by the presence of a salient distractor will also be modulated by the task relevance of the distractor itself (Wood and Wilson, 2010a). Up to now, research on gaze behavior in real-life situations has focused on preparing food (Land et al., 1999; Hayhoe et al., 2003), racket sports (Land and McLeod, 2000; Hayhoe et al., 2012; Mann et al., 2013), walking (’t Hart et al., 2009; Foulsham et al., 2011), and cycling (Zeuwts et al., 2016). Within these tasks, subjects had either to perform pure body movements without object manipulation or to interact with objects in peripersonal space. However, none of these studies considered gaze behavior when subjects are interacting with other persons beyond peripersonal space who will exert an influence on their own performance.

We aimed to investigate gaze localization in a natural environment during the execution of a goal-directed action in the presence of multiple task-relevant objects (ball, target location, and distractor). Based on previous studies and in line with the instruction to shoot as accurately as possible, we expected that penalty takers would direct their gaze predominantly toward the ball. Depending on the experimental conditions, the distractor can be considered as task-relevant (when the goalkeeper tries to save the ball) or task-irrelevant (when the goalkeeper is just standing in the middle of the goal without responding to the shot). To this end, we analyzed where penalty takers direct their gaze during the run-up leading to the final kick while the goalkeeper is trying to interact actively or only trying to distract the penalty taker. This was contrasted with a control condition in which no goalkeeper was present. We focused on the following questions: (a) How does the penalty taker’s gaze behavior change during the run-up? (b) Does the presence (task-irrelevant or task-relevant) or absence of a distractor affect the penalty taker’s gaze behavior? (c) Does the gaze behavior of the penalty taker affect shooting performance?

## Materials and Methods

### Participants

Ten male intermediate-level soccer players (playing experience: 16.6 ± 2.7 years, practice per week: 5.8 ± 1.4 h) aged 18 to 27 years (*M* = 22.0, *SD* = 3.0) participated in this study. Three participants were self-declared left-footers; seven were right-footers. All had normal or corrected to normal vision. The research reported here conformed to the Declaration of Helsinki and was approved by the local Ethics Committee. Before the experiment started, every participant gave written informed consent. All participants were naïve to the aim of the study.

### Experimental Setting

**FIGURE 1:**
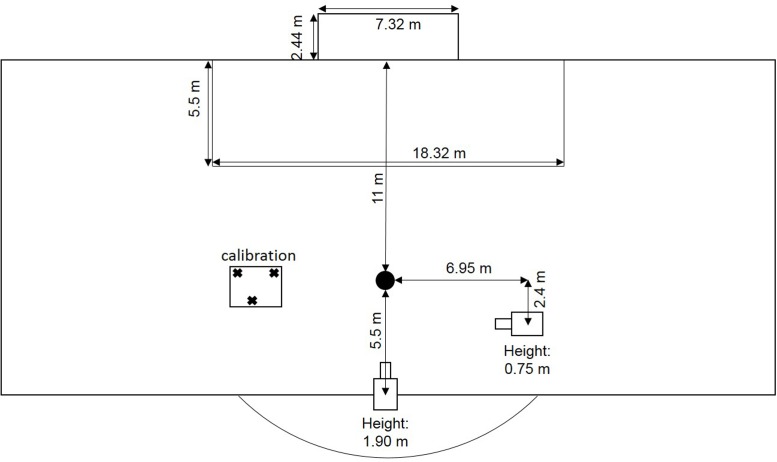
Experimental setup.

Penalties were performed in the natural environment of a soccer pitch (**Figure 1**) complying with international guidelines (Fédération Internationale de Football Association, 2015). The goal size was 7.32 m × 2.44 m and the distance between the penalty mark and the goal was 11 m. Every shot was performed with a standard size 5 football (Pro Touch Force 3000). The same male goalkeeper (age: 26, experience: 16 years) was selected for all trials and all participants.

Run-up was recorded (50 Hz) with a video camera (Panasonic HDC-HS900) placed 7.0 m to the right of the penalty mark when facing the goal and 0.75 m above the ground. Within these videos, we determined foot-ball contact as well as the last step, the second last step, and the third last step before foot-ball contact. Participants were free to choose the duration and the number of steps for their run-up; however, each participant took at least three steps. Furthermore, participants were free to choose the angle of the run-up. These specifications were also in accordance with international guidelines.

Gaze behavior was recorded with a head-mounted mobile binocular eye tracker (SMI iViewETG, SensoMotoric Instruments, Germany) with automatic parallax compensation using iViewETG (Version 2.1) recording software. The environment was recorded by a video camera (960 × 720 pixels) built into the glasses at a frame rate of 30 Hz. Two cameras, also built into the glasses, recorded eye movements at a frame rate of 60 Hz. Data were stored on a mobile recording unit (Samsung Galaxy S4 GT-I9506, Yateley, United Kingdom) placed in a belt bag while performing penalties. After recording, video data were exported to an avi file on a laptop (Lenovo X230, ThinkPad, United States) using BeGaze software (Version 3.5.101).

A second video camera (Panasonic HDC-HS900) placed 5.5 m behind the penalty mark (away from the goal) and 1.9 m above the ground recorded (50 Hz) the scored goals and the ball end location. Screenshots from the videos were taken when the ball passed the goal line or the goalkeeper saved the ball. Afterward, screenshots were analyzed with Matlab (R2015a, MathWorks, Natick, MA, United States) in order to ascertain the horizontal and vertical ball end location in relation to the goal.

Prior to starting the experiment, a three-point calibration was conducted to verify the point of gaze. To avoid head movements during calibration, the chin was fixed on a wooden plane. Calibration was rechecked after each third trial and, if necessary, adjusted. Gaze position accuracy was determined to about 0.5° and the spatial resolution of the system to about 0.1°.

### Design

Participants shot a total of 48 penalties split into three different conditions (16 shots per condition). Within each condition, they had to shoot eight shots to the lower corner and eight shots to the upper corner. Right-footed participants had to shoot either into the bottom right corner or the left upper corner; left-footed participants, into either the bottom left corner or the right upper corner. This arrangement created symmetric conditions for left- and right-footed participants. To examine kinematics and gaze behavior for right-footed and left-footed participants, the data of left-footed participants were mirrored symmetrically. This procedure is not considered as a standard protocol because in most studies only right-footed participants are selected. The three conditions were (1) *no goalkeeper*, (2) *arm waving*, and (3) *reaction*. Conditions were designed to define situations with an increasingly salient distractor along with different modes of the distractor (the goalkeeper). In the condition *no goalkeeper*, the goalkeeper was absent; and in the condition *arm waving*, the goalkeeper was task-irrelevant because he did not show any direct response and did not try to save the penalty. Penalty takers were informed in this condition that they did not have to expect opponents to react and try to save the penalty. The condition *reaction* was most similar to the real-life situation of shooting a penalty. Hence, the goalkeeper was accordingly task-relevant because he tried to save the ball. In contrast to the condition *arm waving*, participants in the condition *reaction* were instructed explicitly that the goalkeeper would try to save the penalty. The sequence of conditions was counterbalanced between participants, and the direction of shots (bottom corner vs. upper corner) was randomized within each participant. Penalty takers and the goalkeeper were aware of the current condition because conditions were performed blockwise and they were informed before each block about the relevant behavior options of the goalkeeper.

### Procedure

Before starting each trial, participants were placed with their shoulders orthogonal to the goal line. Although this condition differed from visual search behavior in an actual penalty, it allowed us to control for visual search behavior before the run-up. Penalty takers were instructed about the corner they should shoot the ball. This instruction was given in such a way that the goalkeeper could not note the following shot direction and was therefore expected to react as naturally as possible. The penalty taker then had to fixate a clapperboard hold by an experimenter. The objective of this procedure was the synchronization of the eye-tracker and the video camera recording the run-up. After operating the clapperboard, the penalty taker was free to take the penalty in his own way.

To standardize the experimental setting, participants were instructed to shoot as accurately as possible. Furthermore, they were asked not to deceive the goalkeeper by looking in one direction and shooting to the opposite side or by slowing down their speed during the run-up. However, we did not instruct the penalty taker to ignore or to consider the goalkeeper. In the condition *no goalkeeper* and *arm waving*, participants were instructed to shoot in the same way as in the condition *reaction*. Our instructions ensured that the penalty taker would use the keeper-independent strategy and try to shoot as accurately as possible, because he selected the target location toward which he was going to aim without considering reactions made by the goalkeeper before the run-up.

When the goalkeeper was present (conditions *arm waving* and *reaction*), he stood centrally in the goal, spreading out his arms and waving them up and down until the penalty taker had shot the penalty. In the condition *reaction*, he was instructed to save as many penalties as possible. He was also instructed to react only when the penalty taker had shot the ball and he did not receive any information about the direction of the forthcoming shot.

### Data Analysis

The penalty taker’s run-up was partitioned into four segments: (1) *preparation phase*: time between the first gaze at an area of interest (AOI) and the third last step (duration: *M* = 2,290 ms; *SD* = 1,071 ms); (2) *third last phase*: time between the third last step and the second last step (duration: *M* = 325 ms; *SD* = 117 ms); (3) *second last phase:* time between the second last step and the last step (duration: *M* = 268 ms; *SD* = 33 ms); and (4) *last phase*: time between the last step and foot-ball contact (duration: *M* = 99 ms; *SD* = 23 ms). We identified step segments manually through a frame-by-frame inspection of the video sequences selecting those frames in which the respective foot made ground contact. The preparation phase was similarly defined as in previous studies and the following three phases are commonly denoted as ‘execution phase’ (e.g., Wood and Wilson, 2010a; Noël and van der Kamp, 2012).

Gaze data were analyzed frame by frame by two observers. Both observers coded five participants each so that they were counterbalanced across participants. To analyze gaze location, AOIs were defined for six different regions: (1) *Ball*: when the gaze was directed toward the ball. (2) *Target area*: when the gaze was directed toward the side to which the penalty taker had to aim. (3) *Middle*: when the gaze was directed toward the middle of the goal (with no goalkeeper present) or when the gaze was directed toward the goalkeeper. (4) *Opposite area*: when the gaze was directed toward the opposite side of the corner to which the penalty taker had to aim. Each section (Target area, Middle, and Opposite area) measured a vertical distance of 2.44 m (from the goal line to the bar) as well as a horizontal distance of 2.44 m (all three sections together extended from goal post to goal post). (5) *Ground*: when the gaze was directed toward the ground (i.e., the surface of the soccer field) between the ball and the goal. Furthermore, we defined a residual category (6) *Other*: all other locations that were considered irrelevant for the task. The AOIs were defined in terms of the objects. In the video a colored circle (30 pixels) indicated the gaze of the subjects. Whenever the circle (gaze) overlapped with an object (Ball, Target area, Middle, Opposite area, Ground) we coded it as the respective object. When the circle (gaze) did not overlap with an object it was coded as Other. The start of each trial was defined as the moment when the gaze of the penalty taker was directed toward one of the following AOIs for the first time after fixating the clapperboard: Ball, Target area, Middle, or Opposite area. The end of a trial was defined by foot-ball contact. A total of 8.6% of gaze data showed missing data due to, for instance, blinking or sunlight. Missing data were excluded from further analyses. Fixations were determined when the gaze was directed continuously toward an AOI for at least seven frames (>100 ms, Vickers, 2007; cf. Land and Tatler, 2009) based on the frame rate of the eye tracker (60 Hz).

**FIGURE 2:**
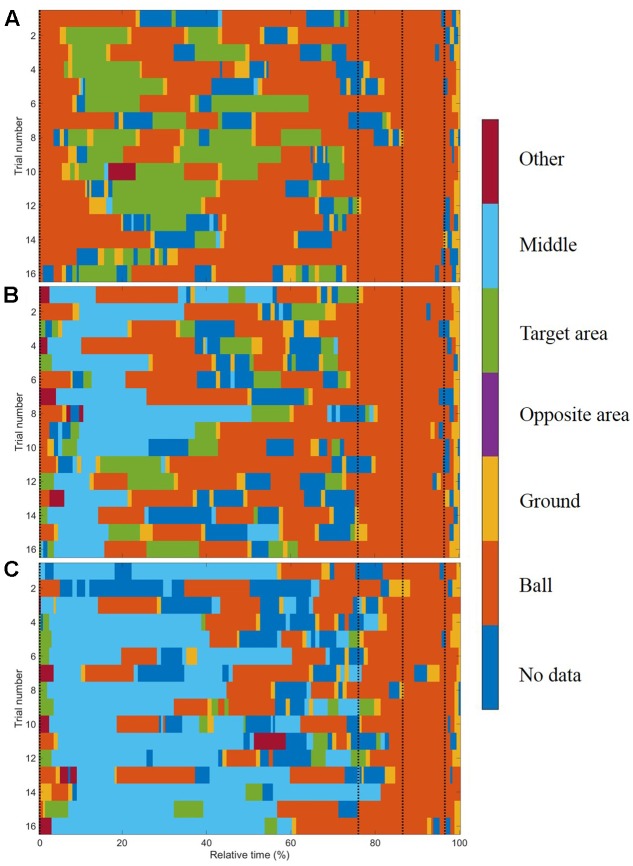
Gaze allocation matrix for one typical subject for conditions **(A)** no goalkeeper, **(B)** arm waving, and **(C)** reaction. The black dashed lines represent the start of the four segments of the run-up: the preparation phase, the third last phase, the second last phase, and the last phase. Each trial’s duration was normalized by its length and then divided into 3,333 ms that was approximately the mean duration of all trials.

**FIGURE 3:**
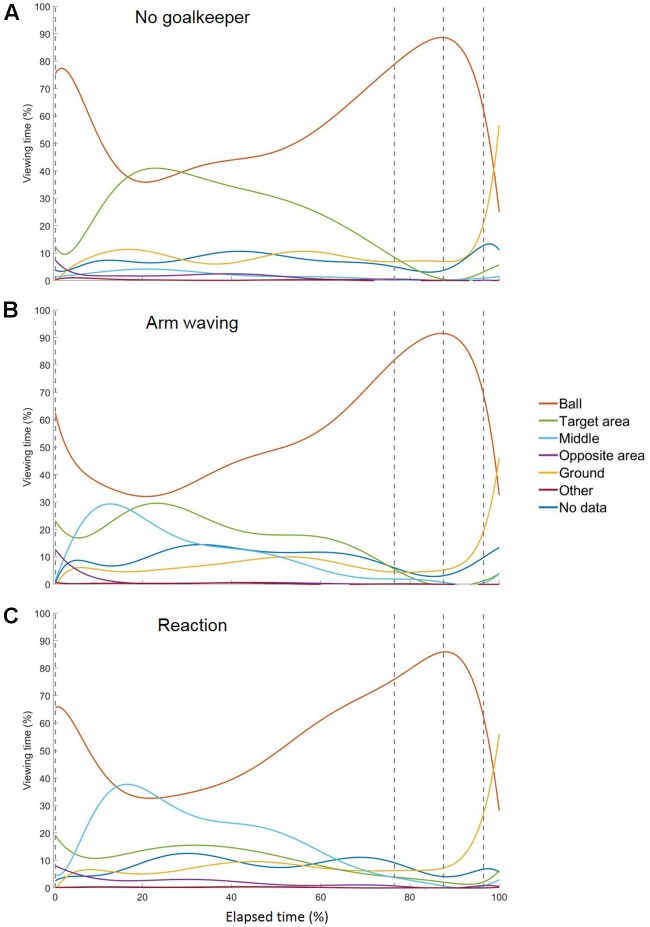
Temporal course of gaze behavior during the run-up for conditions **(A)** no goalkeeper, **(B)** arm waving, and **(C)** reaction. The gray dashed lines represent the start of the four segments of the run-up: the preparation phase, the third last phase, the second last phase, and the last phase. Each trial’s duration was normalized by its length and the divided into 3,333 ms that was approximately the mean duration of all trials.

We computed the following two dependent variables in order to analyze gaze behavior: (1) *Viewing time*: the percentage amount of time the gaze was directed toward each AOI. For a better comparison within and between subjects, we normalized each trial’s duration by its length to 3,333 ms (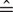 200 frames; based on the frame rate (60 Hz) of the eye tracker). The duration of 3,333 ms was selected because it represents approximately the average duration of all trials. This routine was applied due to different durations for all trials and all phases within and between subjects. For further analysis, we applied percentage values of the normalized data (cf. **Figures 2**, **3**). (2) *Final fixation during the run-up*: the duration as well as the location of the last fixation before foot-ball contact.

Penalty-taking performance was assessed by the number of goals scored and the ball end location in the horizontal direction (Wood and Wilson, 2010a; Timmis et al., 2014). To analyze the ball end location, we took a screenshot at the moment when the ball crossed the goal line. This included trials in which the goalkeeper saved the ball, although they would have counted as a success in the control condition. Trials in which the ball missed the goal were excluded from further analyses (van der Kamp, 2006; Wood and Wilson, 2010a; Noël and van der Kamp, 2012). The bottom center of the goal was defined as origin (0/0). However, it has been identified that shooting tests are not reliable and the internal consistency is low (Ali, 2011; Höner et al., 2015). Therefore, we investigated the internal consistency of the penalty performance in terms of the ball end location. For each condition (no goalkeeper, arm waving and reaction) and for each corner (bottom corner and upper corner), we determined the mean ball end location (horizontal direction) for shot one to four and for shot five to eight. Pearson product correlations between the mean ball end location from shot 1 to 4 and from shot 5 to 8 were first calculated for each combination (condition × corner) separately and then averaged after Fisher *z* transformation. Results showed a mean correlation coefficient of 0.36. This result shows that shooting test are not really reliable which is consistent with findings by Höner et al. (2015). However, ball end location provides the only possibility of analyzing accuracy of penalty performance.

Because we did not find any significant differences in gaze behavior between the bottom corner and the upper corner, we collapsed the gaze data. Viewing times for the AOIs were analyzed with repeated measures ANOVAs (Noël and van der Kamp, 2012; Timmis et al., 2014). However, it must be taken into account that the AOIs are interdependent. When the viewing time of an AOI increases the sum of the viewing times of the other AOIs must decrease and vice versa. However, there does not exist a reciprocal relationship between two variables. Therefore, we decided to report separate ANOVAs for the dependent variables. *Post hoc* comparisons were calculated using *t*-tests with Bonferroni corrections; effect sizes were calculated as partial eta squared, and the significance level was set at 0.05.

## Results

### Viewing Time

In general, subjects showed similar gaze behavior when shooting penalty kicks both within and between experimental conditions. To present interindividual variations, we have provided diagrams for each subject and each condition separately (see Supplementary Material). **Figure 2** presents the gaze allocation matrix for one typical subject, and **Figure 3** presents the mean amount of time gaze was directed toward each AOI for all subjects in the conditions *no goalkeeper*, *arm waving*, and *reaction*. **Figure 3** focuses particularly on where penalty takers directed their gaze during the run-up. Both figures show that, irrespective of condition, penalty takers directed their gaze most of the time toward the ball (65%). In the majority of cases (70%), the first gaze was directed toward the ball followed by a period of time in which gaze was directed toward all AOIs, though, mainly toward the ball, the target area, or the middle of the goal. At the end of the preparation phase, the amount of time the gaze was directed toward the ball increased significantly; and during the last three phases, the gaze was directed almost exclusively toward the ball. To investigate the consistency of gaze behavior for conditions during the run-up we analyzed the number of fixations for each trial. Therefore, we applied an univariate ANOVA with repeated measures for the factor condition (no goalkeeper vs. arm waving vs. reaction). Results showed a significant main effect of condition, *F*_(2,18)_ = 5.00, *p* < 0.05, $\eta_{p}^{2}$ = 0.36. *Post hoc* analyses with Bonferroni corrections revealed significant (*p* < 0.05) less fixations in condition arm waving (number of fixations: *M* = 4.7; *SD* = 1.4) compared to condition reaction (number of fixations: *M* = 5.3; *SD* = 1.6). Condition no goalkeeper (number of fixations: *M* = 5.5; *SD* = 2.2) was not significantly different compared to condition arm waving (*p* = 0.10) and to condition reaction (*p* = 0.99). For the percentage viewing time on the AOIs (ball, target area, middle, ground), we calculated separate 3 (condition: no goalkeeper vs. arm waving vs. reaction) × 4 (phase: preparation phase vs. third last phase vs. second last phase vs. last phase) ANOVAs with repeated measures. **Tables 1**–**3** present *post hoc* analyses (*t*-test with Bonferroni correction) of each area of interest for the main effect of condition, for the main effect of phase and for the Condition × Phase interaction.

**Table 1:** *Post hoc* analyses of the ANOVA for each area of interest.

l				*t*-test with Bonferroni correction	
lAOI	Condition	*Mean* (%)	*SD* (%)	NG	AW
lBall	NG	64.6	15.5		
l	AW	65.9	14.0	ns	
l	R	63.7	10.6	ns	ns
lTarget area	NG	10.3	7.0		
l	AW	8.4	7.6	ns	
l	R	5.6	4.6	∗	ns
lMiddle	NG	1.1	1.3		
l	AW	4.0	3.8	†	
l	R	6.1	4.0	∗∗	†
lGround	NG	15.3	9.7		
l	AW	13.2	8.9	ns	
l	R	16.5	7.9	ns	ns

*Presented are *t*-tests with Bonferroni corrections for the main effects of condition*.

*AOI = area of interest; NG = no goalkeeper; AW = arm waving; R = reaction; ns = *p* ≥ 0.10, ^†^ = *p* < 0.10, ^∗^ = *p* < 0.05, ^∗∗^ = *p* < 0.01, ^∗∗∗^ = *p* < 0.001*.

**Table 2:** *Post hoc* analyses of the ANOVA for each area of interest.

				*t*-test with Bonferroni correction	
AOI	Phase	*Mean* (%)	*SD* (%)	Prep	Third	Second
Ball	Prep	46.2	19.4			
	Third	78.7	18.7	∗		
	Second	86.3	18.2	∗ ∗	ns	
	Last	47.7	30.6	ns	ns	∗∗
Target area	Prep	21.4	16.9			
	Third	8.1	9.6	ns		
	Second	0.6	1.0	∗	ns	
	Last	2.3	5.5	∗	ns	ns
Middle	Prep	12.8	10.2			
	Third	0.4	0.5	∗		
	Second	0.2	0.3	∗	ns	
	Last	1.4	4.3	†	ns	ns
Ground	Prep	7.4	4.0			
	Third	7.0	5.2	ns		
	Second	8.9	17.5	ns	ns	
	Last	36.6	26.6	†	∗	†

*Presented are *t*-tests with Bonferroni corrections for the main effects of phase*.

*AOI = area of interest; Prep = preparation phase; Third = third last phase; Second = second last phase; Last = last phase; ns = *p* ≥ 0.10, ^†^ = *p* < 0.10, ^∗^ = *p* < 0.05, ^∗∗^ = *p* < 0.01, ^∗∗∗^ = *p* < 0.001*.

**Table 3:** *Post hoc* analyses from the ANOVA for each area of interest (AOI).

					*t*-test with Bonferroni correction	
AOI	Phase	Condition	*Mean* (%)	*SD* (%)	NG	AW
Ball	Prep	NG	50.2	19.9		
		AW	44.0	22.4	ns	
		R	44.5	20.7	ns	ns
	Third	NG	77.7	23.0		
		AW	77.4	23.4	ns	
		R	81.0	14.0	ns	ns
	Second	NG	86.1	22.9		
		AW	88.6	17.0	ns	
		R	84.2	16.9	ns	ns
	Last	NG	44.2	33.5		
		AW	53.8	32.3	ns	
		R	45.0	28.7	ns	ns
Target area	Prep	NG	29.3	20.3		
		AW	21.9	20.9	ns	
		R	12.9	11.7	**	ns
	Third	NG	10.1	13.7		
		AW	9.6	12.5	ns	
		R	4.7	4.6	ns	ns
	Second	NG	0.4	1.0		
		AW	0.6	0.2	ns	
		R	1.3	2.7	ns	ns
	Last	NG	1.3	4.1		
		AW	1.9	5.4	ns	
		R	3.5	7.5	ns	ns
Middle	Prep	NG	2.5	2.0		
		AW	13.9	14.5	†	
		R	22.1	16.8	∗∗	†
	Third	NG	0.3	0.9		
		AW	0.3	0.5	ns	
		R	0.6	0.6	ns	ns
	Second	NG	0.1	0.4		
		AW	0.1	0.2	ns	
		R	0.3	0.6	ns	ns
	Last	NG	1.2	3.9		
		AW	1.6	5.0	ns	
		R	1.5	4.1	ns	ns
Ground	Prep	NG	8.4	4.7		
		AW	6.6	3.6	ns	
		R	7.1	4.3	ns	ns
	Third	NG	6.6	5.9		
		AW	7.0	7.1	ns	
		R	7.6	5.1	ns	ns
	Second	NG	9.2	21.8		
		AW	8.0	17.6	ns	
		R	9.7	13.7	ns	ns
	Last	NG	37.2	28.2		
		AW	31.1	27.6	ns	
		R	41.6	27.4	ns	ns

*Presented are *t*-tests with Bonferroni corrections between the conditions of each phase*.

*NG = condition no goalkeeper; AW = condition arm waving; R = condition reaction; Prep = preparation phase; Third = third last phase; Second = second last phase; Last = last phase; ns = *p* ≥ 0.10, ^†^ = *p* < 0.10, ^∗^ = *p* < 0.05, ^∗∗^ = *p* < 0.01, ^∗∗∗^ = *p* < 0.001*.

#### Ball

The ANOVA for the viewing time on the ball showed a significant main effect of phase, *F*_(3,27)_ = 9.70, *p* < 0.001, $\eta_{p}^{2}$ = 0.52. The main effect of condition, *F*_(2,18)_ = 0.48, *ns*, and the Condition × Phase interaction, *F*_(6,54)_ = 1.78, *p* = 0.12, did not attain significance. *Post hoc* analyses with Bonferroni correction for the main effect of phase showed that the gaze was directed significantly longer toward the ball in the third last phase and second last phase compared to the preparation phase and the last phase. The shorter viewing time on the ball during the last phase resulted from a drift of gaze from the ball toward the ground (cf. **Figure 3**) that will be described in more detail below. Hence, the gaze behavior toward the ball seemed to be independent of the presence of a goalkeeper.

### Target Area

Results for the viewing time on the target area showed a significant main effect of phase, *F*_(3,27)_ = 10.27, *p* < 0.01, $\eta_{p}^{2}$ = 0.53, a significant main effect of condition, *F*_(2,18)_ = 6.21, *p* < 0.01, $\eta_{p}^{2}$ = 0.41, and a significant Condition × Phase interaction, *F*_(6,54)_ = 6.68, *p* < 0.01, $\eta_{p}^{2}$ = 0.43. *Post hoc* analyses with Bonferroni correction for the main effect of phase showed that the viewing time on the target area decreased significantly across time—particularly between the preparation phase and the last two phases. *Post hoc* analyses with Bonferroni correction for the main effect of condition showed that the viewing time on the target area decreased with an increasingly salient distractor. However, only the condition *no goalkeeper* and the condition *reaction* differed significantly. *Post hoc* analyses with Bonferroni correction showed that the Condition × Phase interaction resulted from significant differences between conditions in the preparation phase but not in the other three phases. Results showed that in the preparation phase, the viewing time on the target area with an increasingly salient distractor. However, only the condition *no goalkeeper* and the condition *reaction* differed significantly.

#### Middle of the Goal

The ANOVA for the viewing time on the middle of the goal revealed a significant main effect of phase, *F*_(3,27)_ = 11.77, *p* < 0.01, $\eta_{p}^{2}$ = 0.57, a significant main effect of condition, *F*_(2,18)_ = 11.66, *p* < 0.01, $\eta_{p}^{2}$ = 0.56, and a significant Condition × Phase interaction, *F*_(6,54)_ = 10.02, *p* < 0.01, $\eta_{p}^{2}$ = 0.53. *Post hoc* analyses with Bonferroni correction for the main effect of phase revealed that the viewing time on the middle of the goal decreased significantly between the preparation phase compared to the last three phases. However, the last three phases did not differ significantly from each other. *Post hoc* analyses with Bonferroni correction for the main effect of condition showed that the viewing time on the middle increased with an increasingly salient and task-relevant distractor. However, only the condition *no goalkeeper* and the condition *reaction* differed significantly. All other combinations did not attain significance. *Post hoc* analyses with Bonferroni correction for the Condition × Phase interaction showed significant differences between conditions in the preparation phase but not in the other three phases. Results showed that in the preparation phase, viewing time on the middle increased with an increasingly salient distractor. However, only the condition *no goalkeeper* and the condition *reaction* differed significantly.

#### Ground

The ANOVA for the viewing time on the ground revealed a significant main effect of phase, *F*_(3,27)_ = 8.25, *p* < 0.01, $\eta_{p}^{2}$ = 0.48, as well as a significant main effect of condition, *F*_(2,18)_ = 3.89, *p* < 0.05, $\eta_{p}^{2}$ = 0.30. The Condition × Phase interaction, *F*_(6,54)_ = 1.69, *p* < 0.14, did not attain significance. *Post hoc* analyses with Bonferroni correction showed that the main effect of phase resulted from a drift of the gaze from the ball toward the ground during the last phase (cf. **Figures 2**, **3**). This drift was characterized by a continuous shift from the last fixation on the ball toward the target area, whereas the gaze remained stationary on the ground for a short duration. This unexpected phenomenon was observed in 6 out of the 10 participants.

### Duration of the Last Fixation

The duration of the last fixation was analyzed using a univariate ANOVA with repeated measures for the factor condition (no goalkeeper vs. arm waving vs. reaction). Results did not reveal a significant main effect of condition, *F*_(2,18)_ = 3.96, *p* = 0.07, but a linear decrease in the duration of the last fixation (no goalkeeper: 740 ± 390 ms vs. arm waving: 683 ± 326 ms vs. reaction: 625 ± 298 ms). Participants selected only the three AOIs ball (*M* = 77.9%), ground (*M* = 20.4%), and target area (*M* = 1.7%) for their last fixation. A chi-square test revealed a significant preference for selecting the ball as the location of the last fixation, χ^2^_(2,_ *_N_* = _480)_ = 454.65, *p* < 0.001, *w*^2^ = 0.95.

### Penalty Performance

Two dependent variables were introduced to assess the effect of the experimental conditions on penalty performance. First, the number of actual goals was analyzed using chi-square tests. Performance was significantly better, χ^2^_(1,_ *_N_* = _480)_ = 28.68, *p* < 0.001, for the bottom corner (*M* = 88.8%) than for the upper corner (*M* = 68.8%). However, no significant differences were found between conditions, χ^2^_(2,_ *_N_* = _480)_ = 0.52, *ns* (no goalkeeper: *M* = 79.4% vs. arm waving: *M* = 76.9% vs. reaction: *M* = 80.0%). Second, ball end location (**Table 4**) was analyzed in terms of the absolute horizontal distance from the origin at the bottom center of the goal. A 2 (corner: bottom corner vs. upper corner) × 3 (condition: no goalkeeper vs. arm waving vs. reaction) ANOVA with repeated measures for both factors was applied to analyze the absolute horizontal distance. Results showed no significant differences for either the main effect of corner, *F*_(1,9)_ = 3.69, *p* = 0.08, the main effect of condition, *F*_(2,18)_ = 1.20, *p* = 0.32, or the Corner × Condition interaction, *F*_(2,18)_ < 1, *ns*. It has to be considered, that the reliability of shot tests has been proven to be unsatisfactory.

**Table 4:** Absolute horizontal distance between ball end location and the origin at the bottom center of the goal.

	No goalkeeper		Arm waving		Reaction	
	Bottom corner	Upper corner	Bottom corner	Upper corner	Bottom corner	Upper corner
Horizontal distance (cm)	259.5 (19.4)	271.1 (44.3)	264.4 (24.3)	282.0 (23.1)	252.7 (36.8)	275.9 (30.9)
Scored goals	73	54	71	54	69	57
Missed shots	7	26	9	26	6	21
Saved shots	n/a	n/a	n/a	n/a	5	2

*The number of scored shots, the number of missed shots, and the number of saved shots are presented for each condition (*N* = 160). Absolute horizontal distances are reported as means with standard deviations in parentheses*.

## Discussion

In the present study, our aim was to examine where penalty takers direct their gaze during the execution of this far-aiming task with multiple task-relevant objects (ball, goal, and goalkeeper). Furthermore, we controlled for the presence/absence of a salient distractor and manipulated its task relevance. The goalkeeper was task-relevant (condition *reaction*) when he tried to save the ball and task-irrelevant (condition *arm waving*) when he was just standing in the middle of the goal without responding to the shot. Penalty takers were informed about the goalkeeper’s reactions. Particularly the penalty takers were informed about trials, when the goalkeeper tried to save the penalty. We then assessed the selection of fixation targets during the execution of soccer penalty shots. This task consisted of a proximal target (the ball), a distal target (the respective corner of the goal), and a task-specific distractor (the goalkeeper).

All in all, our results confirm that the timing of gaze during ongoing interactions with the environment employs a “just-in-time” heuristic (Ballard et al., 1995) in that gaze behavior is tuned dynamically to task-specific locations (Hayhoe and Ballard, 2005). Therefore, gaze behavior concerning the AOIs is interdependent, an issue which has to be kept in mind. Although participants’ gaze was focused mainly on the proximal target in our task, they did direct their gaze toward the distal target for a significant amount of time during the early phase of the run-up (cf. **Figure 3**). Thus, the different proportions of viewing time for the proximal and the distal target can be explained as small deviations in relation to task specificity, because foot-ball contact at the proximal target would produce larger deviations from the distal target. Furthermore, it seems to be necessary to explore the environment during the early phase of the run-up (Rothkopf et al., 2007) in order to perform a spatial calibration. With respect to the modulation of gaze behavior by the presence of the distractor during the late run-up, results show that task specificity can indeed inhibit and even switch off automatic fixations on salient stimuli. More specifically, we found this inhibition to depend on the time course of the action: inhibition was strongest in the vicinity of foot-ball contact when fixating the proximal target was of maximum importance, whereas inhibition was relaxed with increasing temporal distance from foot-ball contact. In line with the primate of successful task performance, the presence of the distractor and the resulting modulatory influence on gaze behavior during early phases of the run-up had no effect on the quality of action execution in terms of shooting accuracy. In the following, we shall discuss each of these findings in more detail.

### How Does the Penalty Taker’s Gaze Behavior Change during the Run-up?

During the run-up, gaze was directed predominantly toward the ball irrespective of the distractor. Furthermore, the amount of time gaze was directed toward the ball increased before foot-ball contact. This shows that for penalty takers using the keeper-independent strategy, gazing at the ball seems to be most important to attain an optimal foot-ball contact.

During the preparation phase, the penalty taker’s gaze was directed toward the ball, the target area, or the middle and, for some parts, the opposite area and the ground. Gaze behavior in the preparation phase was driven presumably by the penalty taker’s need to explore the environment at the beginning of the attempt in order to generate a spatial representation of it (Land et al., 1999; Hayhoe et al., 2003; Rothkopf et al., 2007). This means that penalty takers had to analyze the exact position of the ball and update their representation of the goal and the goalkeeper (when he was present) before making decisions about the following movements (Tong et al., 2017).

During the last three phases, penalty takers directed their gaze toward the ball and only rarely toward the distal target (**Figures 2**, **3**). Therefore, an early update of the distal target seems to be task-adequate, and the exact representation of the goal is instrumental in planning the shot direction. Evidently, the most relevant AOI was identified for the proximal target ball. This solution to the motor problem of allocating attention to the most significant affordance seems to be adequate, because the distal target can be retrieved from memory following an occasional recalibration of the far target. Concerning task-specific objects with high visual salience, Rothkopf et al. (2007) have shown that objects with a high visual salience (here the goalkeeper, the goal, and the ball) are fixated at the beginning of a trial.

The mean duration of the last fixation before foot-ball contact was 683 ms irrespective of the experimental conditions, and the most frequently observed AOI was the ball (approximately 80% of trials). Similar gaze behavior has been reported not only for penalty taking (Wood and Wilson, 2010b, 2011; Noël and van der Kamp, 2012) but also for grasping (Johansson et al., 2001). These authors assumed that an early fixation on an object occurs before manipulation in order to perform a spatial calibration that can be useful for motor planning—in our case, to hit the ball in an optimal way. This gaze behavior is described as “pro-active” (Hayhoe and Ballard, 2005); that means, the eyes are directed toward a location at which an event is expected—in our case, foot-ball contact. Other studies have demonstrated that the time between the first gaze toward an object and contact with that object ranges from about 500 ms (Land et al., 1999) to about 900 ms (Brouwer et al., 2009). The last fixation on an object seems to depend on which information is necessary for the task (Hayhoe and Ballard, 2005). Hence, we suppose that the last fixation was directed toward the ball, because the instructions requested a highly accurate foot-ball contact, and the main source of the necessary information for a successful performance was the ball. Although results regarding the duration of the last fixation on the ball showed only marginally significant differences, we argue that the duration of the last fixation did not decrease with the higher salience of the distractor (the goalkeeper) because of a further need for visual processing, but because shorter durations reflected some kind of allocation of attention toward the distal target and the goalkeeper. Additionally, we found that gaze drifted from the ball toward the ground during the last phase. In this study, we did not run further analyses of this phenomenon, though future research should take this into account and take a closer look at whether this pattern of gaze behavior can predict the corner toward which penalty takers are aiming.

### Does the Presence or Absence of a Task-Specific Distractor Affect the Penalty Taker’s Gaze Behavior?

Our results indicate that when goalkeepers are present, penalty takers are not able to ignore them completely during the preparation phase even though they are task-irrelevant (condition *arm waving*). We suggest that this pattern of gaze behavior appears to be due to the salience of the goalkeeper, and the fact that penalty takers are accustomed to seeing a goalkeeper between the goalposts. Penalty takers try to identify (voluntarily or involuntarily) the position of the goalkeeper relative to the center of the goal or the goalkeeper’s posture. Previous studies (Masters et al., 2007; van der Kamp and Masters, 2008) have shown that goalkeepers rarely stand exactly in the center of the goal. Masters et al. (2007) have reported that penalty takers shoot the ball mostly to the side with the greater open area (59%). Additionally, penalty takers try to pick up information from goalkeepers’ movements indicating in which direction they will dive (van der Kamp et al., 2011). In real-world situations, these information sources seem to play a major role in successful penalty taking. When a goalkeeper is present but task-irrelevant (condition *arm waving*), penalty takers direct their gaze more toward the target area during the preparation phase. In contrast, when the goalkeeper is task-relevant (condition *reaction*) and tries to save the ball, penalty takers avoid directing their gaze toward the target area during this preparation phase. We argue that the target area did not change its relevance between the conditions *arm waving* and *reaction*, although the amount of time the penalty takers directed their gaze toward the target area in the condition *reaction* decreased compared to the condition *arm waving*. A speculative assumption is that the penalty takers wanted to conceal which corner they were going to shoot toward, and instead of looking toward the target area, they directed their gaze toward the goalkeeper. This is consistent with findings reported by Dicks et al. (2010) showing that during the early phase of the run-up, goalkeepers direct their gaze toward the head of the penalty takers to obtain information on where they will shoot the ball. Another argument why penalty takers direct their gaze during the early run-up to either the target area (condition *no goalkeeper*), the target area and the middle (condition *arm waving*), or the middle area (condition *reaction*) could be that one of these locations (target area and middle) is sufficient to calibrate the environment.

In contrast to the preparation phase, during the last three phases, penalty takers showed a similar pattern of gaze behavior irrespective of condition. They directed their gaze mostly toward the ball; and, during the last phase, toward the ground. This result indicates that penalty takers were able to ignore the goalkeeper, the goal, and other locations almost completely (cf. **Figures 2**, **3**), because respective locations were less important (Johansson et al., 2001; Rothkopf et al., 2007). This supports the finding that the required information (optimal foot-ball contact) is obtained immediately when it is needed, and that the gaze is directed toward locations that are best for performing the specific task (shooting as accurately as possible) and not toward the objects/locations that are salient (Johansson et al., 2001; Land and Hayhoe, 2001; Rothkopf et al., 2007). In addition, the mechanisms responsible for the salience are “off duty” while performing a specific task (Schütz et al., 2011). We suggest that this pattern of gaze behavior is the best strategy for shooting as accurately as possible. To achieve this goal, the penalty taker has to minimize the deviation of the ball end location. Therefore, an optimal foot-ball contact seems to be especially important, and we suggest that this is the reason why gaze was directed almost exclusively toward the ball in our study. This included not only the point in time when the ball was shot but also the run-up when an optimal timing of gait had to be organized (Lee et al., 1982). This subjective interpretation of a penalty taker’s strategy might be underlined by the fact that, for biomechanical reasons alone, goalkeepers have no chance of saving a top-seeded shot, however hard they try. This argument is supported by results from the condition *no goalkeeper* in which no goalkeeper was present who tried to save the ball. However, penalty takers still directed their gaze toward the ball instead of looking toward the target area. In addition, other studies (Wood and Wilson, 2010b, 2011; Noël and van der Kamp, 2012) also found that when penalty takers try to shoot as accurately as possible, they direct their gaze mainly toward the ball during the last 600 ms before foot-ball contact. Wood and Wilson (2010b, 2011) supposed that this gaze behavior is necessary to generate motor commands resulting from visual information. They also suggested that the location of the last fixation is more important than the duration of this fixation or any other location of fixation. We suggest that during the last three phases when using the keeper-independent strategy, penalty takers used a well-developed visual routine (Hayhoe, 2000) learned at an early age (Lees and Nolan, 1998) and habitualized over the years (Hoppe and Rothkopf, 2016). This visual routine has become so stabilized that it is resistant to external influences (e.g., movements made by the goalkeeper) or to the success/failure of a shot. A further argument why penalty takers direct their gaze toward the ball could be that—in contrast to the opposite-independent strategy—they do not want to give information to the goalkeeper about where they are going to shoot. Thus, they want to hide their gaze. However, studies on the gaze behavior of goalkeepers in soccer (Savelsbergh et al., 2002; Dicks et al., 2010) and in ice hockey (Panchuk and Vickers, 2006) have shown that goalkeepers direct their gaze either toward the body, the legs, the ball, or the ground around the ball. Dicks et al. (2010) found that goalkeepers directed their gaze exclusively toward the ball or the legs 1,000 ms before foot-ball contact until foot-ball contact. Thus, it does not seem to be necessary to hide the gaze, particularly during the late run-up, because goalkeepers try to obtain other information from the kinematics of the penalty taker.

### Does the Gaze Behavior of the Penalty Taker Affect Shooting Performance?

Our data indicate that a distracting stimulus (absent vs. task-irrelevant vs. task-relevant) has hardly any impact on the performance of the penalty taker while using the keeper-independent strategy. This can be explained on the basis of the gaze behavior during the run-up. Our results indicated differences in performance (number of scored goals) only between the bottom and the upper corner. We suggest that shooting to the bottom corner is easier, because penalty takers just have to direct the ball in the horizontal direction (left/right). In contrast, shooting to the upper corner is more difficult due to the need to direct the ball in both the horizontal (left/right) and the vertical (up/down) direction. As a result, they have to deal with more degrees of freedom. Furthermore, results for shots that miss a goal are congruent with other findings (Wood and Wilson, 2010a; Navarro et al., 2013) indicating that the number of shots that miss the goal depends on the penalty taker’s skill rather than on the presence of a goalkeeper. However, it has to be mentioned that possibly due to our instructions (to shoot as accurately as possible) penalty takers’ main goal was not only to score a goal. Thus, the penalty takers’ task could be interpreted as a motor precision task, like for example a free-throw in basketball. Taken this interpretation into consideration, the goalkeeper did not provide relevant information which could be used by the penalty taker to complete the motor precision task. In this case, the goalkeeper did only influence the final result of the task (goal or no goal). It could be argued that penalty takers’ main goal was not only to score a goal but also to be as accurate as possible because in the condition *no goalkeeper* penalty takers failed to score a goal for about 20%, irrespective of the corner. This shows that penalty takers tried to be precise instead of just scoring a goal. Furthermore, Wood and Wilson (2010a) reported that shots were more centralized with a moving goalkeeper (standing in the middle of the goal, waving arms up and down) compared to shots with a stationary goalkeeper (standing in the middle of the goal, arms by the side). However, we were unable to replicate this finding in the present study. We suggest that—as mentioned before—penalty takers fall back on a visual routine developed over the long term, especially for the late phases of the run-up.

## Conclusion

We analyzed gaze direction during the execution of a far-aiming task with multiple task-relevant objects (ball, goal, and goalkeeper). The present findings are manifold: first, throughout the run-up, gaze was directed most of the time toward the ball irrespective of condition. This suggests that the ball was most important for shooting as accurately as possible, because penalty takers had to attain an optimal foot-ball contact. Second, although the distractor impacted on the gaze behavior of the penalty taker, the gaze behavior was influenced only during the early phase of the run-up. In the late phases of the run-up, the distractor did not affect gaze behavior. Third, performance of the penalty takers was not influenced by the absence or presence of a distractor (absent vs. task-irrelevant vs. task-relevant), because penalty takers directed their gaze toward the ball during the last three phases. These results indicate that whether gaze is driven by salience of the stimulus setting or by attentional demands depends on the phase of the task in which the penalty taker is engaged.

## Author Contributions

JK made substantial contributions to conception and design, acquisition of data, and analysis and interpretation of data. MH and JM made substantial contribution to conception and design, and analysis and interpretation of data. JK, MH, and JM participated in drafting the article and revising it critically for important intellectual content; and give final approval of the version to be submitted and are accountable for all aspects of the work in ensuring that questions related to the accuracy or integrity of any part of the work are appropriately investigated and resolved.

## Conflict of Interest Statement

The authors declare that the research was conducted in the absence of any commercial or financial relationships that could be construed as a potential conflict of interest. The reviewer AP and handling Editor declared their shared affiliation, and the handling Editor states that the process nevertheless met the standards of a fair and objective review.
