# Supplementary material for: Gaze Behavior in a Natural Environment with a Task-Relevant Distractor: How the Presence of a Goalkeeper Distracts the Penalty Taker
Source: Front Psychol. 2018 Jan 26;9:19. doi: 10.3389/fpsyg.2018.00019 (PMC5790805; doi:10.3389/fpsyg.2018.00019)
Supplement: Supplementary file 1 [file Data_Sheet_1.DOCX]

Supplementary Material

Gaze behavior in a natural environment with a task-relevant distractor: How the presence of a goalkeeper distracts the penalty taker

Kurz Johannes *, Hegele Mathias, Munzert Jörn

*** Correspondence:** Kurz Johannes: Johannes.kurz@sport.uni-giessen.de

# Supplementary Figures


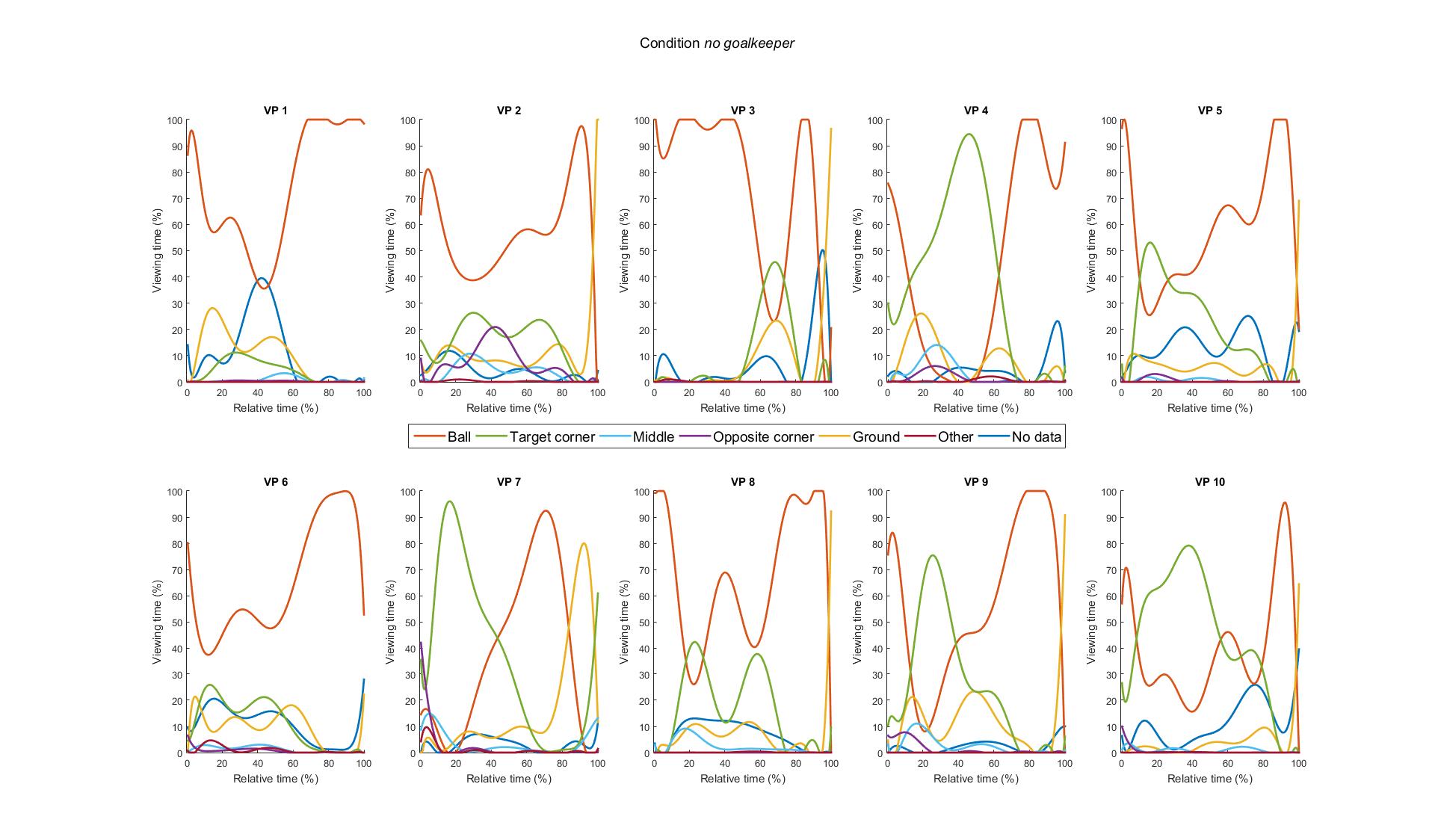


**Supplementary Figure 1.** Temporal course of gaze behavior during the run-up for condition no goalkeeper for each subject. The gray dashed lines represent the start of the four segments of the run-up: the preparation phase, the third last phase, the second last phase, and the last phase. Each trial was normalized in time to a frame rate of 200 frames (3,333 ms) that was approximately the mean duration of all trials.


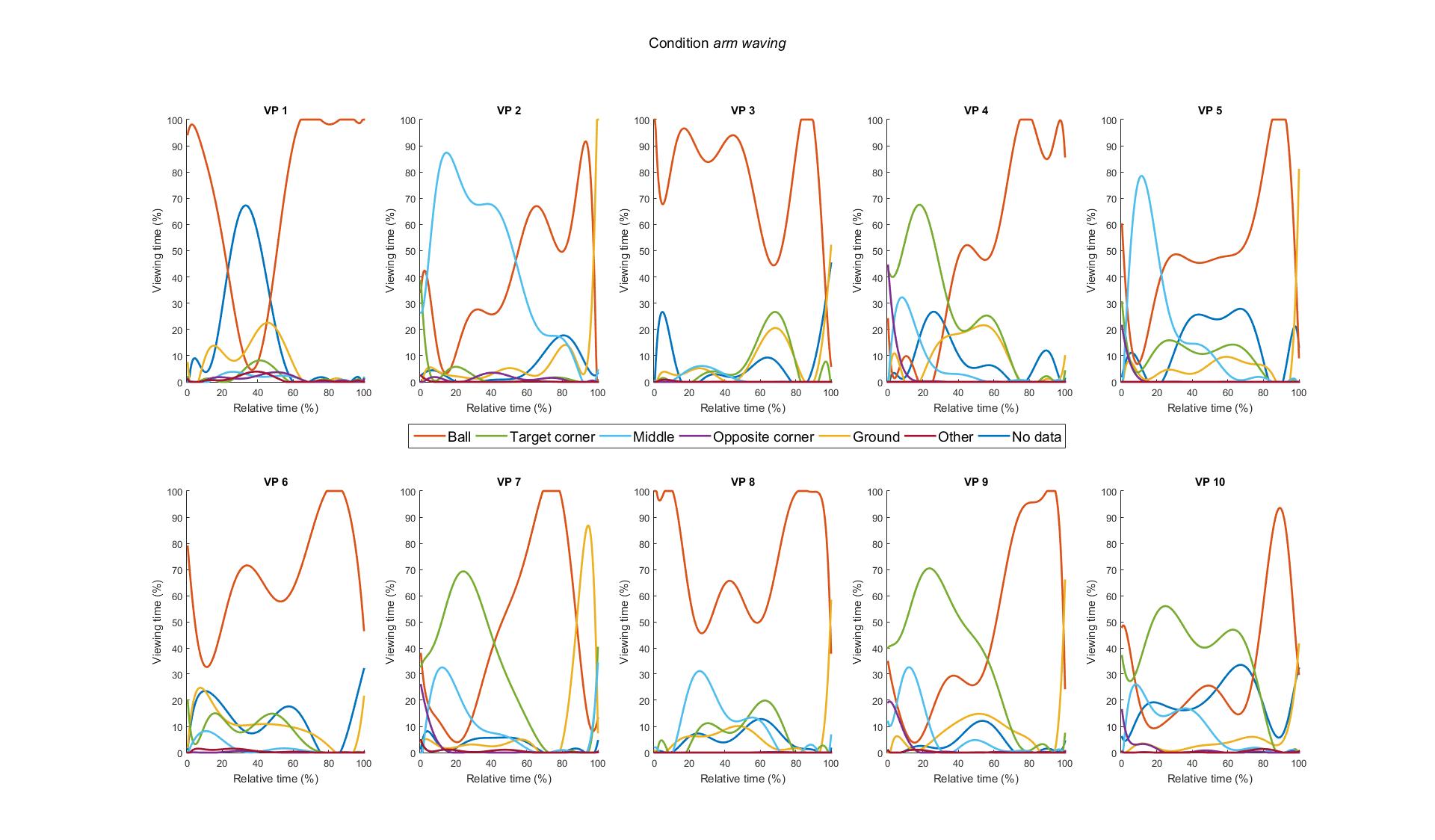
**Supplementary Figure 2.** Temporal course of gaze behavior during the run-up for condition arm waving for each subject. The gray dashed lines represent the start of the four segments of the run-up: the preparation phase, the third last phase, the second last phase, and the last phase. Each trial was normalized in time to a frame rate of 200 frames (3,333 ms) that was approximately the mean duration of all trials.


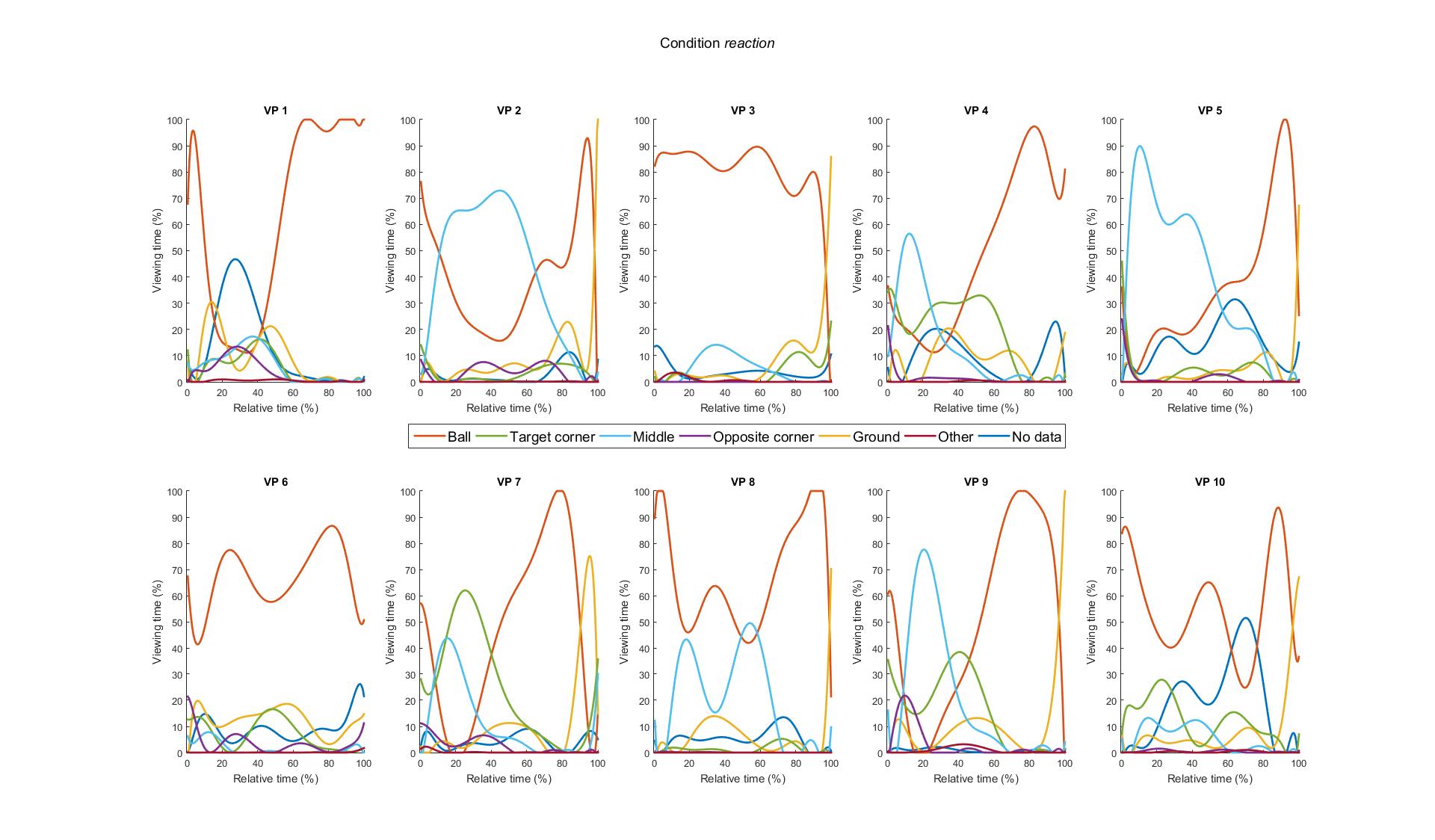


**Supplementary Figure 3.** Temporal course of gaze behavior during the run-up for condition reaction for each subject. The gray dashed lines represent the start of the four segments of the run-up: the preparation phase, the third last phase, the second last phase, and the last phase. Each trial was normalized in time to a frame rate of 200 frames (3,333 ms) that was approximately the mean duration of all trials
